# Multivariate Patterns in Object-Selective Cortex Dissociate Perceptual and Physical Shape Similarity

**DOI:** 10.1371/journal.pbio.0060187

**Published:** 2008-07-29

**Authors:** Johannes Haushofer, Margaret S Livingstone, Nancy Kanwisher

**Affiliations:** 1 Department of Neurobiology, Harvard Medical School, Boston, Massachusetts, United States of America; 2 Department of Brain and Cognitive Sciences, and McGovern Institute for Brain Research, Massachusetts Institute of Technology, Cambridge, Massachusetts, United States of America; National Institute of Mental Health, National Institutes of Health, United States of America

## Abstract

Prior research has identified the lateral occipital complex (LOC) as a critical cortical region for the representation of object shape in humans. However, little is known about the nature of the representations contained in the LOC and their relationship to the perceptual experience of shape. We used human functional MRI to measure the physical, behavioral, and neural similarity between pairs of novel shapes to ask whether the representations of shape contained in subregions of the LOC more closely reflect the physical stimuli themselves, or the perceptual experience of those stimuli. Perceptual similarity measures for each pair of shapes were obtained from a psychophysical same-different task; physical similarity measures were based on stimulus parameters; and neural similarity measures were obtained from multivoxel pattern analysis methods applied to anterior LOC (pFs) and posterior LOC (LO). We found that the pattern of pairwise shape similarities in LO most closely matched physical shape similarities, whereas shape similarities in pFs most closely matched perceptual shape similarities. Further, shape representations were similar across participants in LO but highly variable across participants in pFs. Together, these findings indicate that activation patterns in subregions of object-selective cortex encode objects according to a hierarchy, with stimulus-based representations in posterior regions and subjective and observer-specific representations in anterior regions.

## Author Summary

As early as 1031 a.d., the Arab scholar Ibn al-Haytham suggested that visual experience was not veridical, but inherently subjective. During the last few decades, this observation has given rise to one of the core questions in visual neuroscience: how does the subjective experience of visual stimuli relate to their neural representations in the brain? It is well-known that visual shape is represented in a brain region called lateral occipital complex (LOC). However, do these representations reflect physical or perceptual stimulus characteristics? We presented observers with a set of complex visual stimuli and obtained three measures of similarity for these stimuli: a physical similarity measure based on stimulus parameters; a behavioral similarity measure based on discrimination performance; and finally a neural similarity measure based on multivariate pattern analyses in LOC. We found that in anterior LOC, neural stimulus similarities correlated with subjective perceptual similarities, but not with physical stimulus similarities; the reverse was true in posterior LOC. In addition, neural similarities were consistent across participants in posterior LOC, but highly variable across participants in anterior LOC. Together these findings suggest a two-part answer to the question of how cortical object representations relate to subjective experience: anterior regions appear to contain subjective, individually variable shape representations, whereas posterior regions contain stimulus-based shape representations.

## Abstract 3

How does the subjective experience of visual shapes relate to the neural representations of these shapes in the brain? Using psychophysics, functional MRI, and multivariate pattern analysis methods, this study shows that activation patterns in anterior, shape-selective brain regions reflect perceptual shape similarities, whereas patterns in posterior regions reflect physical similarities.

## Introduction

What is the neural code for object shape? This question has been at the core of systems neuroscience for decades. In monkeys, inferotemporal (IT) cortex has been shown to contain cells selective for complex shapes [1]; in humans, functional magnetic resonance imaging (fMRI) has identified a brain region known as lateral occipital complex (LOC) as a neural center for object representation [2,3]. This region responds more to intact than scrambled images of everyday objects [2,3] and is thought to be critical for object recognition [4,5]. However, the nature of the representations in these object-selective regions remains poorly understood.

A number of previous studies suggest that the coding of objects in high-level visual cortex may reflect subjective perceptual experience of shapes. For instance, LOC adapts across changes in low-level physical stimulus properties that leave perceived shape unaltered, but not across changes that affect perceived shape [6,7]. Furthermore, the fMRI signal in LOC tracks recognition performance more accurately than activation in retinotopic cortex [5,8], and both IT neurons and the fMRI signal in LOC reflect the perceptual similarity of stimuli [8,9]. Finally, Kayaert et al. [10,11] found that IT cells are more strongly modulated by perceptually salient stimulus changes (nonaccidental properties) than by metric changes of equal physical magnitude.

FMRI studies of visual processing have traditionally focused on mean activation levels, looking for brain regions showing a difference in activation between different stimulus conditions. More recent studies, in contrast, have illustrated the importance of the distributed pattern of activation in representing information about stimulus conditions [12–14]. Haxby et al. [12] first showed that even when there is no difference in the mean activation levels of specific conditions across occipitotemporal cortex, object category can still be determined from the distributed pattern of activation using a correlation method. Recently, Williams et al. [8] demonstrated that activation patterns contain object-specific information only on trials where recognition is successful. This finding raises the question whether activation patterns contain detailed information about subjective visual experience.

We used a combination of human fMRI and psychophysics to test the hypothesis that distributed activation patterns in LOC reflect perceived shape. We created a novel artificial shape space, in which physical similarity was controlled by gradual, parametric changes in aspect ratio and skew. Perceptual similarity was measured by psychophysical discrimination performance between the shapes, and neural similarity was measured by the correlations between the fMRI activation patterns of these shapes in LOC. (Note that we use the term “neural” to refer to fMRI activation patterns because of the high correlation between the BOLD signal and neuronal activity [15]) We found significant correlations between neural and perceptual similarity measures in LOC. Interestingly, this finding was restricted to the anterior portion of LOC (pFs); in the posterior portion (LO), we found significant correlations between neural and physical similarity measures. In addition, neural similarities were consistent across participants in posterior LOC, but highly variable across participants in anterior LOC. Together, these results suggest that object representations in posterior LOC reflect the physical stimulus, while representations in anterior LOC reflect subjective shape experience.

## Results

**Figure 1:**
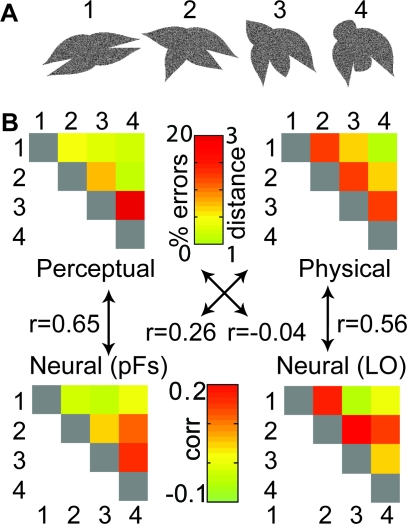
Stimuli and Analysis Method (A) The Stimuli used in the fMRI and behavioral experiments. Adjacent stimuli were equidistant in the aspect ratio/skew stimulus space, and together the four stimuli formed a straight line through this space. We could thus control physical similarity in terms of aspect ratio and skew. Area was kept constant across stimuli. (B) Example of perceptual (top left), physical (top right), and neural (bottom) similarity matrices, together with correlation coefficients between the matrices. These correlation coefficients were obtained for all participants and are summarized in Figure 2.

We created a novel artificial stimulus space consisting of four complex objects (Figure 1A). Each stimulus had four radially arranged protrusions (two half-parabolas joined at the vertices), which varied parametrically in aspect ratio and skew across stimuli. This stimulus space had five important features. First, IT cortex contains cells that are tuned to aspect ratio and skew, independently of one another [16]; thus, our stimuli varied along dimensions likely to be relevant in object-selective areas. Second, the four shapes used in the experiment were equidistant in aspect ratio and skew, and we thereby controlled important aspects of the their physical similarity; we refer to these aspects of similarity as “physical similarity,” while noting that other definitions of physical similarity are possible (see below for an analysis using a V1-like measure of similarity). Third, the stimuli were novel, allowing us to investigate shape similarities without confounds from semantic or learned associations. Fourth, spatial fMRI activation patterns in LOC have recently been shown to contain information sufficient for discrimination of such novel shapes [8,14,17]. Finally, the stimuli were chosen such that perceptual similarities correlated somewhat with physical similarity, but not perfectly, leaving room for the neural similarities to correlate, e.g., with perceptual similarity without necessarily also correlating with physical similarity, and vice-versa.

**Figure 2:**
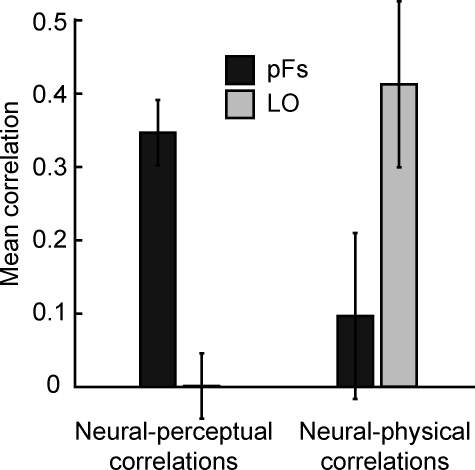
Mean Correlations across Participants between Neural and Perceptual Similarities (Left) and Neural and Physical Similarities (Right) Neural-perceptual correlations are high in pFs and low in LO, and the reverse is true for neural-physical correlations. Shown are means ± 1 (conventional) standard error (SE).

To study the relationship between perceptual, physical, and neural similarities in pFs and LO, we obtained three similarity measures for each pair of stimuli as follows. First, for each of the six possible pairs of nonidentical stimuli, physical similarity was measured by the inverse pairwise distances of the four shapes in the aspect ratio/skew space (Figure 1B, top right panel). As pointed out above, aspect ratio and skew were chosen because these dimensions are thought to be of relevance in high-level visual cortex [16]. Since the four stimuli formed a continuum with equal distances between adjacent stimuli, the six possible pairs of the four stimuli had distances of 1, 1, 1, 2, 2, and 3 steps; these distances were converted to similarities by inverting their values, to yield the similarity values 3, 3, 3, 2, 2, and 1 (see below for a different measure of physical similarity).

Second, to obtain a measure of perceptual shape similarity, we conducted a separate behavioral experiment outside the scanner with the same participants. On each trial, two shapes were shown in succession, and participants responded whether the two shapes were identical or different. Each shape was shown for 17 ms, with a forward and a backward mask of 50 ms each (without gaps between stimulus and masks), followed by a 1,500 ms response period. The proportion of trials on which a particular participant responded “identical” to a pair of stimuli that were in fact different was used as a measure of the perceptual similarity of that pair of stimuli. An example of a perceptual similarity matrix is shown in Figure 1B (top left panel). Note that we use the confusion rate merely as a proxy for true, first-person perceptual similarity, and do not wish to argue strongly that two stimuli that are confused with high probability necessarily also have highly similar qualia. Our definition of perceptual similarity is therefore merely an operational one in the context and for the practical purposes of this experiment.

Finally, to obtain a measure of neural similarity, we scanned the brains of eight participants using fMRI. Since we had a specific hypothesis about neural coding in object-selective cortex, we first identified the human object-selective region LOC in an independent localizer scan, using the standard comparison of intact versus scrambled everyday objects [2] (*p* < 10^−4^). LOC can be subdivided into a posterior portion, LO, on the lateral surface of occipitotemporal cortex; and an anterior portion, pFs, on the fusiform gyrus of the temporal lobe [18]. These two anatomically distinct portions of LOC were defined as separate regions of interest (ROIs). The ROI approach is of advantage because it is not subject to multiple comparisons problems.

In separate scans, we presented participants with the four shapes, using an event-related design. Each stimulus was shown for 300 ms, followed by a blank period of 1,700 ms, during which the participants had to respond whether the current stimulus was identical to that on the previous trial (one-back task). The purpose of this task was to keep participants' attention focused on the stimuli. We then extracted the spatially distributed activation patterns of each individual stimulus from the two LOC ROIs, on a voxel-by-voxel basis. Thus, in each participant and for each ROI, we obtained four vectors, each representing the voxelwise activation pattern of one particular shape in that ROI. We then computed the correlations between each of the six pairs of activation patterns for nonidentical stimuli, separately for pFs and LO. This resulted in six correlation coefficients for each ROI, one for each possible pair of the four shapes. An example of a neural similarity matrix is shown in Figure 1B (bottom panels).

### Correlations between Neural and Perceptual/Physical Similarity in LOC

Thus, we obtained physical, perceptual, and neural similarity measures for each possible pair of stimuli. We next compared these six-element similarity matrices to one another, by computing their correlation coefficients within participants and ROIs (Figure 1B). A high correlation between, e.g., the perceptual similarity matrix and the neural similarity matrix in a given ROI would indicate that if two stimuli are similar perceptually, they are also similar neurally in that ROI, i.e., their neural activation patterns are highly correlated with one another. Our hypothesis predicted that neural and perceptual similarity should be correlated in LOC.

The results confirmed this hypothesis, with an interesting twist. Neural and perceptual similarities were positively correlated in pFs, with an average correlation of 0.35 across participants, whereas in LO the average neural-perceptual correlation was only 0.001 (Figure 2). Conversely, neural and physical similarities were strongly positively correlated in LO (average correlation 0.41), but much more weakly in pFs (average correlation 0.10; Figure 2).

To quantify these results, we initially applied the Fisher z transformation to all correlation coefficients. This method transforms the non-normally distributed correlation coefficients into normally distributed variables, which allows the use of standard analysis of variance methods [19] (for details, see Materials and Methods). Statistical analysis after Fisher z transformation confirmed that across participants, the correlation coefficients between neural and perceptual similarities were significantly greater than zero in pFs (*t*(5) = 5.66, *p* < 0.001), but not greater than zero in LO (*t*(5) = 0.10, *p* = 0.38). Conversely, the correlations between neural and physical similarities were significantly greater than zero in LO (*t*(5) = 2.66, *p* < 0.05), but not in pFs (*t*(5) = 0.70, *p* = 0.29). A two-way analysis of variance (ANOVA) with region of interest (pFs versus LO) and correlation type (neural-perceptual versus neural-physical) as factors revealed a significant interaction of ROI and correlation type (*F*(1,5) = 13.79, *p* < 0.005), confirming the dissociation between these ROIs: neural pattern similarities in pFs correspond to subjective shape similarities, while neural pattern similarities in LO correspond to physical shape similarities.

These results were not due to differential mean signal levels for any of our stimuli, for two reasons: first, the correlation analysis does not take into account mean levels of activation; second, there were no differences in mean signal between the four stimuli in either region of interest (pFs: *F*(3,21) = 1.86, *p* = 0.17; LO: *F*(3,21) = 0.17, *p* = 0.92). Moreover, these results cannot be due to task performance, since critically the perceptual similarity measure was obtained in a separate testing session, while in the scanner participants performed an easy one-back task. (A control analysis for potential effects due to this task is reported below.)

### Inter-Participant Reliability

**Figure 3:**
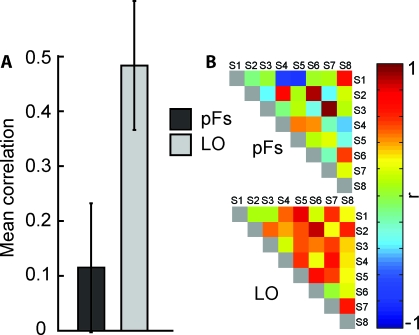
Inter-Participant Reliability (A) Mean neural inter-particiapnt correlation coefficients in pFs (left) and LO (right). Consistent with observer-specific, subjective similarities in pFs, and physically-based similarities in LO, inter-participant reliability is low in pFs and high in LO. Shown are means ± 1 (conventional) SE. (B) Neural inter-particiapnt reliability in pFs (top) and LO (bottom). Each cell represents the correlation coefficient between the neural similarity matrices of two individual participants. A high correlation indicates that pairs of stimuli that are neurally similar in one participant are also neurally similar in the other participant.

As a further test of this finding, we speculated that if neural similarities in pFs reflect subjective perceptual similarities, the correspondence of neural similarities across participants in this region might be low: if a given pair of stimuli is neurally similar in pFs in one participant, the same pair may be neurally different in another participant whose subjective percept is different. Conversely, if neural similarities in LO reflect physical similarities, they should not differ greatly across participants. In other words, in pFs we would expect low inter-participant reliability of the neural similarities, whereas in LO we would expect high inter-participant reliability. To test this hypothesis, we correlated the neural similarity matrices of all individual participants with one another, separately for each ROI. This resulted in two 8 × 8 matrices, where each cell represents the correlation between the neural similarity matrices of two individual participants in one ROI (Figure 3). A high correlation in a given cell indicates that in these two participants, the stimulus pairs that are neurally similar in one participant are also neurally similar in the other participant.

As predicted, inter-participant reliability was low in pFs (mean across-participant correlation: 0.07; not different from zero, *t*(25) = 0.92, *p* = 0.26; Figure 3), but high in LO (mean: 0.42; greater than zero, *t*(25) = 7.83, *p* < 0.00000005; Figure 3). The difference between these two ROIs was significant (*t*(25) = −2.80, *p* < 0.05).

Note that this analysis is independent of the results described above: whether the neural similarities in pFs correlate across participants (as tested here) does not depend on whether they correlate with the behavioral similarities within participants (tested above).

### Further Regions of Interest

**Figure 4:**
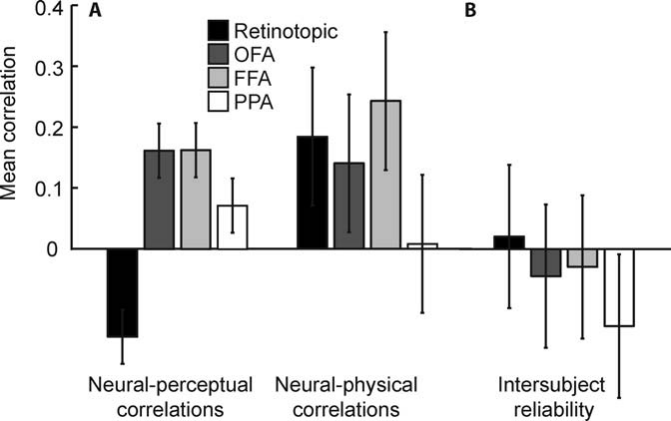
Correlations in Control ROIs (A) Mean correlations across participants between neural and perceptual similarities (left) and neural and physical similarities (right) in control ROIs. No bar is significantly different from zero using Fisher-z–transformed correlations and standard errors. Shown are means ± 1 (conventional) SE. (B) Neural inter-participant reliability in control ROIs. Each bar represents the average correlation coefficient between the neural similarity matrices of all pairs of individual participants. A high correlation indicates that pairs of stimuli that are neurally similar in one participant are also neurally similar in the other participant. No bar is significantly different from zero. Shown are means ± 1 (conventional) SE.

To test whether the results described above are specific to object-selective cortex, we defined a set of further regions of interest: a retinotopic ROI based on activation at the occipital pole during the localizer task, as described before [8]; the fusiform face area (FFA [20]; see also [7]), and the occipital face area (OFA; [21,22]), based on the standard functional contrast of faces against objects (*p* < 10^−4^); and the parahippocampal place area, PPA [23], based on the standard contrast of scenes against objects (*p* < 10^−4^). In none of these regions did the neural similarities exhibit significant correlations with either perceptual or physical similarity (Figure 4A). Moreover, we found no significant inter-participant reliability in any of these regions (Figure 4B). With the exception of PPA, each of these regions contained at least as many voxels as pFs, ruling out the possibility that this finding is due to an inability to detect a correlation in small datasets. However, this possibility remains for PPA, which contained significantly fewer voxels than pFs. Note that FFA did not overlap with pFs in any of our participants.

### Behavioral Results

The psychophysical same-different task used outside the scanner to obtain a measure of perceptual similarity was made as difficult as possible by presenting stimuli for extremely short durations (17 ms each) and using both forward and backward high-energy noise masks (50 ms each). Nevertheless, the performance level was high, with an average of 93% ± 1% correct performance on the same-different task. However, this level of performance corresponds to an average of 44 ± 9 errors over the course of the psychophysical experiment; this number of errors, distributed over the four stimuli used, proved sufficient to obtain a reliable measure of perceptual similarity. In support of this claim, the inter-participant reliability of perceptual similarity was on average *r* = 0.28 across participants (different from zero: *t*(25)=2.77, *p* < 0.05), showing that pairs of stimuli that a particular participant confused with high probability were also perceptually similar for the other participants. However, at the same time the fact that this correlation was not extremely high leaves room for subjective, observer-specific patterns of perceptual similarities.

The average correlation between the physical and perceptual similarity measures across participants was *r* = 0.38; this value was significantly different from zero (*t*(5) = 2.75, *p* < 0.05). Thus, perceptual similarity correlated with physical similarity, but not perfectly, again leaving room for the neural similarity measures to correlate with either the perceptual or the physical similarities without necessarily also correlating with the other.

The one-back task participants performed in the scanner was designed purely to keep participants' attention focused on the stimuli and was very easy to perform; none of our participants made a single mistake on this task. However, to nevertheless control for any possible effects of performance in the scanner, we analyzed the reaction times during the task in the scanner as follows. Since the order of stimulus presentation was randomized using m-sequences, each stimulus was preceded equally often by each other stimulus; therefore, each of the six possible discriminations among the four stimuli entered the neural pattern an equal number of times, and differential performance on any of these conditions could therefore not influence the neural pattern. However, it is possible that the task was easier for some stimuli in general; such a difference would enter the neural pattern of those stimuli and could thus potentially bias our results. Indeed, we observed a significant difference in the reaction times of stimuli 1 and 3 (mean reaction time [RT], stimulus 1: 602 ms ± 13 ms; stimulus 3: 624 ms ± 14 ms; *t*(7) = 8.11, *p* < 0.001), and stimuli 1 and 2 (mean RT, stimulus 2: 619 ms ± 17 ms; *t*(7) = 2.88, *p* < 0.05). To assess the effect of this difference, we used the reaction time differences among the four stimuli during the task in the scanner as a new behavioral similarity measure within each participant; e.g., if a particular participant took an average of 635 ms to respond to stimulus 1, and 649 ms for stimulus 2, the new behavioral similarity of these stimuli would be the negative reaction time difference, i.e. −14 ms (negative to turn the measure from a “difference” into a “similarity” measure). We then re-computed the correlations between the neural similarities and this new behavioral similarity measure. None of the resulting correlations were significant in any of our ROIs, although there was a nonsignificant trend towards a correlation between neural similarities and the new reaction time similarities in LO (mean *r* = 0.33, *t*(5) = 1.75, *p* = 0.09).

### Robustness to Subsampling

**Figure 5:**
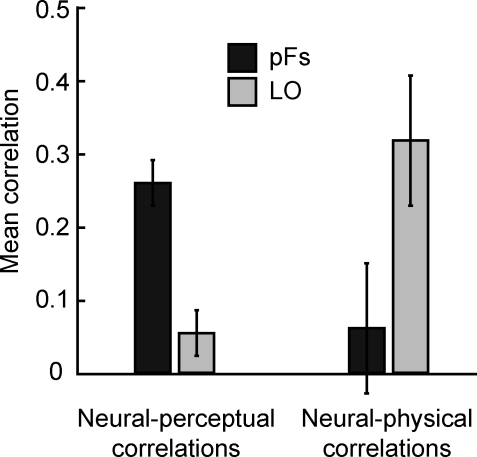
Robustness of Within-Participant Analysis to Subsampling Mean correlations across participants between neural and perceptual similarities (left) and neural and physical similarities (right), after random exclusion of 50% of voxels and 100-fold bootstrapping. Neural-perceptual correlations are high in pFs and low in LO, and the reverse is true for neural-physical correlations. Shown are means ± 1 (conventional) SE.

**Figure 6:**
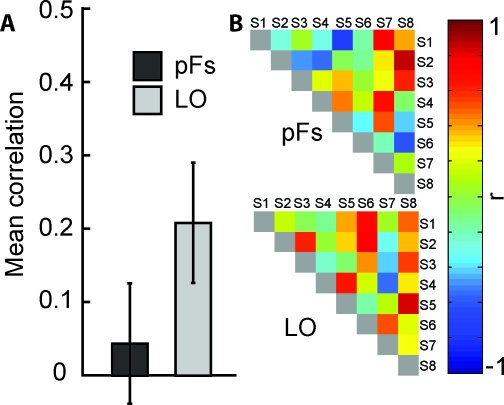
Robustness of Inter-Participant Reliability to Subsampling (A) Mean neural inter-participant correlation coefficients in pFs (left) and LO (right), after random exclusion of 50% of voxels and 100-fold bootstrapping. Consistent with observer-specific, subjective similarities in pFs, and physically-based similarities in LO, inter-participant reliability is low in pFs and high in LO. Shown are means ± 1 (conventional) SE. (B) Neural inter-participant reliability in pFs (top) and LO (bottom), after random exclusion of 50% of voxels and 100-fold bootstrapping. Each cell represents the correlation coefficient between the neural similarity matrices of two individual participants. A high correlation indicates that pairs of stimuli that are neurally similar in one participant are also neurally similar in the other participant.

As pointed out above, it is conceivable in principle that differential numbers of voxels in different ROIs affect the likelihood of detecting correlations. Indeed, LO contained more voxels on average than pFs (pFs: mean 127 ± 36 voxels, LO: mean 287 ± 107 voxels). We therefore wished to test whether the results described above depend on the number of voxels in each ROI. To this end, we conducted a control in which we randomly excluded 50% of the voxels of each ROI. This procedure was repeated 100 times, and an average correlation estimate was obtained by averaging over the 100 bootstrapping iterations. The results were the same as in the main analysis reported above (Figures 5 and 6): the average correlation between neural and perceptual similarities in pFs was 0.25 (different from zero: *t*(5) = 6.32, *p* < 0.001; Figure 5), but that between neural and physical similarities in this region was only 0.05 (not different from zero: *t*(5) = 0.56, *p* = 0.32); in contrast, LO exhibited a significant correlation between neural and physical similarities (mean *r* = 0.32, *t*(5) = 2.33, *p* < 0.05; Figure 6), but not between neural and perceptual similarities (mean *r* = 0.06, *t*(5) = 0.56, *p* = 0.32). The interaction was again significant (*F*(1,5) = 12.00, *p* < 0.005). Similarly, the inter-participant reliability was again high in LO (mean *r* = 0.21, *t*(25) = 2.82, *p* < 0.05), but low in pFs (mean *r* = 0.04, *t*(25) = 0.60, *p* = 0.33; Figure 6). The difference between LO and pFs was significant (*t*(25) = 2.04, *p* = 0.05). Note, however, that subsampling reduced the inter-participant reliability in LO by a factor of one-half (mean *r* = 0.42 to mean *r* = 0.21). In light of this change, and the fact that the average sizes of pFs and LO differed by a factor greater than two, we repeated the subsampling for the inter-participant reliability analysis using not 50% of voxels, but instead equalizing voxel numbers across the two ROIs. Specifically, we excluded random subsets of voxels from the larger ROI, until its size matched that of the smaller ROI, again with 100-fold bootstrapping. The results were comparable to that of the initial analysis: the inter-participant reliability was high in LO (mean *r* = 0.31, *t*(25) = 7.79, *p* < 0.0001), but low in pFs (mean *r* = 0.07, *t*(25) = 0.91, *p* = 0.26), with a significant difference between LO and pFs (*t*(25) = 2.65, *p* < 0.05). Thus, our results are independent of the size of our ROIs.

### An Alternative Physical Similarity Measure

The physical similarity measure reported above was based on the distances of the stimuli from each other in terms of aspect ratio and skew parameters. The high correlation of these aspect ratio/skew distances with neural similarities in LO is consistent with the proposal that LO encodes stimuli in terms of aspect ratio and skew, as has been reported previously for high-level visual cortex in monkeys [16,24]. This finding suggests that LO might no longer correlate with physical similarity if it was defined in a different fashion. As a test of this hypothesis, we replaced the aspect ratio/skew distance measure with an alternative physical similarity measure designed to mimic the properties of area V1: the images were convolved with a set of Gabor filters with orientation and spatial frequency selectivities similar to those found in V1 [25] (see Materials and Methods); the resulting filtered images were then compared for pixelwise similarity. The resulting mean physical similarity matrix correlated well (*r* = 0.67) with the physical similarity measure reported above, i.e., closeness of the stimuli in parameter space. However, the neural similarities of pFs and LO showed no correlation with this V1-type physical similarity measure: in pFs, the mean correlation of the neural similarities with the mean Gabor similarity measure was *r* = 0.06 (not different from zero: *t*(5) = 0.42, p = 0.34); in LO, it was *r* = 0.07 (*t*(5) = 0.45, *p* = 0.34). In addition, we repeated this analysis for each individual Gabor filter (4 orientations × 5 spatial frequencies); none of the resulting 20 correlations between neural and physical similarities were significantly different from zero across participants in either pFs (correlations ranging from −0.09 to 0.10, none significant across participants) or LO (correlations ranging from −0.11 to 0.16, none significant across participants). Thus, the correlation of neural and physical similarities in LO appears to be specific to the case when the physical similarities are described in terms of aspect ratio and skew [16].

The neural patterns in retinotopic cortex only showed a weak correlation with the physical stimulus distances based on this V1-type similarity measure; the mean correlation between neural and Gabor similarities in retinotopic cortex was *r* = 0.15, which did not differ from zero across participants (*t*(5) = 0.76, *p* = 0.27). Moreover, none of the correlations were significant when the individual physical similarity matrices resulting from each of the 20 Gabor filters were correlated one-by-one with neural similarities (correlations ranging from 0.12 to 0.17, none significant across participants). This result is probably due to the fact that the images were presented with a random jitter of ∼2 degrees during scanning, which likely resulted in sufficiently nonoverlapping activations in retinotopic cortex to disrupt the neural similarity estimates in this region, and therefore also any correlation between neural and physical similarities. In support of this hypothesis, the stimuli were not distinguishable in retinotopic cortex using Haxby's pattern discrimination method [12] (mean percent correct discrimination: 44% ± 5%), while they were easily discriminable in pFs (66% ± 4% correct) and LO (65 ± 3% correct; for more details see next section).

### Stability of Neural Patterns across Split-Halves

The results reported above indicate that the neural patterns in our regions of interest contain fine-grained information about perceptual and physical stimulus similarity. These analyses were based on correlations between the neural activation patterns of pairs of stimuli; this measure controls for noise in the data because the formula for the correlation coefficient includes a division by the standard deviations of the data vectors. However, we additionally wished to confirm with a conventional analysis method that these neural patterns were indeed stable and contained information about stimulus identity. To this end, we applied the widely used technique of Haxby et al. [12]: we extracted the activation patterns separately for even and odd runs, and compared “within” and “between” correlations. The mean “within” correlations were 0.10 ± 0.05 and 0.04 ± 0.02 in pFs and LO, respectively, while the mean “between” correlations were 0.04 ± 0.02 and −0.001 ± 0.04 for pFs and LO, respectively. These correlations were low because we used an event-related design. Importantly, however, the “within” correlations were significantly higher than the “between” correlations, indicating that the patterns contained enough information to discriminate between same versus different stimuli (*F*(1,7) = 3.67, *p* < 0.05, two-way ANOVA with ROI and within/between as factors). As a further test of pattern discriminability, we computed the “Haxby Index” [8,12,13,26]. This index estimates classification performance between pairs of stimuli based on the within and between correlations, where 50% is chance performance and 100% is optimal performance. Discrimination performance was 66% ± 4% in pFs and 65% ± 3% in LO; these levels of performance were significantly above chance (pFs: *t*(7) = 4.20, *p* < 0.005; LO: *t*(7) = 4.55, *p* < 0.005). Thus, the patterns in both ROIs were stable enough across the split halves to successfully discriminate between our stimuli.

## Discussion

In sum, we have found that distributed activation patterns in human object-selective cortex contain information about the subjective perceptual similarities between complex visual stimuli. Specifically, we show a dissociation between neural coding of perceptual versus physical similarities within LOC: using independent measures of neural, perceptual, and physical similarity on our set of novel artificial shapes, we find that the neural similarities of shapes in anterior LOC (pFs) correlate with their perceptual similarities. Conversely, the neural similarities in posterior LOC (LO) correlate with the physical similarity of the shapes in the stimulus space. Furthermore, the agreement across participants of the neural similarities is high in LO, but low in pFs, consistent with a physically based representation in LO and a representation based on observer-specific subjective shape experience in pFs.

These results are specific to object-selective cortex, i.e., the regions LO and pFs; additional ROIs including retinotopic cortex, FFA, OFA, and PPA did not show significant correlations between either neural and perceptual or physical similarities. Moreover, the results did not depend on the number of voxels in each ROI.

Our findings confirm previous studies showing that object representations in the ventral stream reflect subjective perception [5–9,11,27], and extend them by showing that the distributed pattern of activation in LOC contains information about idiosyncratic perceptual similarities on a fine-grained scale [14].

Similarly, the finding that posterior LOC shows a correlation between neural and physical stimulus similarity confirms previous studies that have shown selectivity for physical shape features of moderate complexity in high-level visual cortex. In area V4, single-cell and fMRI studies have demonstrated tuning to contour curvature in monkeys [24,28,29] and selectivity for radial and concentric gratings [30] and intermediate-complexity object parts [31] in humans. Single cells in monkey IT cortex have been shown to be tuned to metric changes in simple geometrical shapes [10,16] and to particular combinations of simple shapes [1]. Moreover, IT responses are sensitive to low-level visual properties such as object size, position, and viewpoint [32–34].

Putting the results from pFs and LO together, our findings are consistent with previous evidence regarding an anterior-posterior functional subdivision within LOC: Grill-Spector et al. [35] showed that pFs exhibits more location- and size-invariance than LO; Lerner et al. [36,37] found that pFs was more vulnerable to object scrambling than LO; and Kourtzi et al. [38] showed that pFs does not adapt across changes that alter an object's subjective appearance (convex versus concave), while LO does. Together, these studies suggest that object representations in pFs are more high-level, abstract, and closer to subjective perception than those in LO. Our results substantiate this claim by showing directly that neural similarities correlate with perceptual similarities in pFs but not LO, while neural similarities correlate with physical similarities in LO but not pFs. In contrast to these previous studies, our experiment shows the correspondence between perceptual and neural similarities directly and within individual participants, by using participant-specific measures of perceptual and neural similarity. Furthermore, by computing these neural similarities on the activation pattern across individual voxels, we obtain a richer and more informative measure of neural similarity than can be achieved by averaging the activation across the entire ROI [12].

However, it should be noted that other recent studies have found evidence highlighting the informativeness and behavioral relevance of neural activation patterns in LO: Eger et al. [14] showed that support vector classification within categories was better in LO than pFs; Williams et al. [8] found that correct versus incorrect recognition was reflected in the activation patterns of LO but not pFs. Thus, pFs may not always be the seat of conscious shape perception; instead, the cortical regions whose representations are most closely associated to subjective shape perception may vary with the stimulus, task, and viewing conditions [8].

Three previous studies have shown correlations between pattern information and subjective perception in object-selective cortex. First, Edelman et al. [9] used multi-dimensional scaling (MDS) to uncover the perceptual and neural similarities of a set of categorized stimuli, and found that both the behavioral and the neural measures of similarity followed the stimulus category boundaries (e.g., four-legged animals versus cars). However, their stimulus set consisted of photographs of familiar, every-day objects from a restricted set of categories; thus, it is unclear whether the correspondence between the neural and behavioral measures is due to low-level visual similarity of objects in the same category, category membership itself, or even matching semantic associations of stimuli in the same category. By using novel objects from a parametrized stimulus space we can disentangle perceptual from physical similarity. Moreover, we show a functional dissociation between the two subregions of LOC, which were not analyzed independently in the Edelman et al. [9] study.

Second, Op de Beeck et al. [27] showed in monkeys that the firing rates of neurons in IT cortex reflect perceptual similarities in a set of complex stimuli. The present experiment was inspired by this study, and we replicate its findings in a different species (human) and using a different technique (fMRI). In addition, we show that human object-selective cortex contains both physically based and perceptually based similarity metrics, organized in a posterior-anterior hierarchy.

Third, Williams et al. [8] used a pattern analysis approach similar to the one used here to show that the activation pattern in LOC contains information sufficient for stimulus discrimination only if the participant successfully categorizes the stimulus. This study is similar to ours in that it establishes a link between the distributed activation patterns in LOC and behavioral performance. However, the question it addresses is substantially different from ours: Williams et al. asked whether the information contained in the LOC activation patterns correlated with successful object categorization; in contrast, we ask whether the activation patterns in LOC reflect physical stimulus similarities or subjective perceptual similarities. This difference is also the likely cause for a further one, namely that Williams et al. find correspondence between neural patterns and task performance in LO, but not in pFs, while we show correlations between neural and perceptual similarities in pFs but not in LO (see also [14]). In addition, Williams et al. used visually highly distinctive stimulus categories, whereas the differences between our stimuli were more subtle. Together these differences suggest that coarse category membership [8] and more fine-grained similarity (this study) may be neurally distinguishable at the level of LOC.

In conclusion, our results indicate that object shape may be coded in terms of physical features in posterior LOC, and in terms of subjective shape experience in anterior LOC. Of course, this claim simply replaces one puzzle with another: “What is the neural code for object shape?” is transformed into the equally difficult question, “What are the determinants of subjective shape experience?” However, the advantage of this new question is that it has been the subject of intensive study since the time of the Gestalt psychologists [39], and the accumulated evidence is a rich source of new hypotheses about the neural code for object shape. Combining behavioral measures with fine-grained fMRI pattern analysis methods [8,12,13] may prove a powerful means of solving the puzzle of visual object recognition.

## Materials and Methods

### Participants.

We recruited eight participants from the MIT Human Subject Pool. Each participant was compensated US$60. The study was approved by the MIT Committee on the Use of Humans as Experimental Subjects (COUHES). All participants gave informed consent.

### Stimuli.

Localizer scans: The LOC was localized as the region that responded more strongly to grayscale images of intact objects than to images of scrambled objects (*p* < 10^−4^), as described previously [2,6]. The FFA [20] and the OFA [21,22] were defined as the regions responding more to faces than objects (*p* < 10^−4^). The PPA [23] was defined as the region responding more to scenes than objects (*p* < 10^−4^). The retinotopic ROI was defined based on activation at the occipital pole in a contrast between all stimulus conditions versus baseline in the localizer scans (*p* < 10^−4^; [8]).

*Experimental scans and behavioral experiment*: Four novel stimuli, each measuring 10 degrees across, were used for the experimental scans. The use of novel stimuli ensured that correlations were not due to semantic associations with the stimuli; this was a potential confound in previous pattern similarity studies [9]. Furthermore, we wished to use shape features that are likely to be encoded in object-selective cortex. Single-cell studies have shown that aspect ratio and skew are two such features [10,16]; we therefore created our stimuli based on parametric changes in aspect ratio and skew. Specifically, each stimulus consisted of four protrusions arranged radially around a central disk. Each protrusion was composed of two adjoining half-parabolas of the form *y* = *a x^n^*. The parameters *a* and *n* could be used to vary the skew and aspect ratio of each protrusion parametrically. In doing so, the total area of the stimuli was always kept constant to avoid low-level confounds. We defined aspect ratio as the ratio of the height to the base width of each protrusion, and skew as the position of the vertex with respect to the center of the base; for instance, 0% skew indicates a vertex directly above the center of the base, skew of 100% indicates a vertex directly above the right end of the base, and skew of −100% indicates a vertex directly above the left end of the base. From the left to the right end of the stimulus spectrum, the aspect ratio of the second and fourth protrusions (counting clockwise, beginning at 12 o'clock) decreased by 1.4 and 1.6 on each morph step, respectively; the aspect ratio of the first and third protrusions was fixed. For skew, the first, second, and fourth protrusions moved counterclockwise by 60%, 24%, and 24% for each step, respectively (where a cumulative skew change greater than 100% simply meant moving the vertex of the protrusion beyond its base); the skew of the third protrusion changed in the clockwise direction by 25% on each step. Thus, the four stimuli used were equidistant in terms of aspect ratio and skew, forming a straight line in the stimulus space. The magnitude of the parametric distances between the stimuli was chosen based on informal testing to be at the same time discriminable and not too obvious. The stimuli were filled with random dots, with a mean luminance of 50%, to ensure activation throughout the ventral visual stream. In addition, a chair and a face were included in the stimulus set, to prevent adaptation in ventral visual cortex due to the high similarity among the novel shapes.

### Procedure.

*fMRI experiment*: Each participant was run in one session of about 2 h, consisting of eight experimental scans and four LOC localizer scans. Stimuli were presented using the Psychophysics Toolbox [40] and Matlab (Mathworks).

The localizer scans were run as described previously [6,20,23].

The experimental scans were event-related, and each scan contained 144 stimulus trials and 36 fixation trials. On each trial, one of the six possible stimuli (four novel shapes, one face, one chair) was presented at the center of the screen for 300 ms, followed by a 1,700-ms response period during which participants indicated whether the current stimulus was identical or different from the previous one. The purpose of this task was to keep participants' attention focused on the stimuli. The order of stimulus presentation was optimized using m-sequences (Optseq). Each stimulus occurred 24 times per scan, resulting in a total of 192 times for the whole experiment.

Behavioral experiment: In a separate behavioral session that followed the fMRI experiment with a delay of at least 1 wk, each of the original participants performed a same-different task on pairs of the same four shapes, plus the face and the chair, that were presented in the fMRI experiment. On each trial, two stimuli were shown sequentially, for 17 ms each, with a forward and a backward mask (consisting of a full screen noise field of random letters with high density and overlap) of 50 ms each, followed by a 1,500-ms response period. Perceptual similarities were obtained by computing the proportion of trials on which a particular pair of different stimuli was erroneously considered “identical”. Participants performed 630 trials total. Each stimulus appeared with equal probability on each trial, and with equal probability as the first and second stimulus of each pair.

### Functional imaging.

fMRI scanning was performed on a 3T Siemens Trio Scanner (Siemens) at the Athinoula A. Martinos Center for Biomedical Imaging at the McGovern Institute for Brain Research at MIT. A Gradient Echo single-shot pulse sequence was used (TR = 2 s; TE = 30 ms). Twenty-five slices were collected with a 12-channel head coil. Slices were oriented roughly perpendicular to the calcarine sulcus and covered most of the occipital and posterior temporal lobes, as well as some of the inferior parietal lobes. Slices were 2 mm thick, with a 10% gap, and had an in-plane resolution of 1.6 × 1.6 mm.

### Data analysis.

Data analysis was performed using FS-FAST (http://surfer.nmr.mgh.harvard.edu), fROI (http://froi.sourceforge.net), and custom-written software. Before statistical analysis, images were motion corrected [41], and the data from the blocked localizer scans (not the event-related scans) were smoothed (3 mm full width at half maximum Gaussian kernel).

The LOC was defined as the set of contiguous voxels in the central occipitotemporal cortex that showed significantly stronger activation (*p* < 10^−4^, uncorrected) to intact objects than to scrambled versions of the same objects [2]. Two subregions of LOC were defined as ROIs, as described previously [42]: a posterior portion, LO, on the lateral surface of occipitotemporal cortex; and an anterior portion, pFs, on the fusiform gyrus of the temporal lobe [18]. Furthermore, we defined four control regions of interest: the FFA [20] and the OFA [21,22], based on the standard functional contrast of faces against objects (*p* < 10^−4^); the PPA [23], based on the standard contrast of scenes against objects (*p* < 10^−4^); and a retinotopic ROI based on activation at the occipital pole in a contrast between all stimulus conditions versus baseline in the localizer scans (*p* < 10^−4^; [8]). The FFA did not overlap with pFs in any of our participants, as is sometimes the case.

For the blocked localizer scans, statistical maps were calculated by correlating the signal time course with a gamma function (delta = 2.25, tau = 1.25) for each voxel convolved with the block timecourse. For the event-related scans, the hemodynamic response was extracted using a deconvolution analysis, without any assumptions about the shape of the response. The peaks of the fMRI responses of each of the four novel shapes were extracted from each ROI, for all voxels separately. This resulted in four patterns per ROI, each representing the distributed activation pattern to a particular stimulus in that ROI. Neural similarities were obtained by computing the Pearson correlation coefficient between these patterns, as described above.

To assess the statistical significance of the correlation matrices results, we first applied the Fisher z transformation to the data and then performed *t*-tests and ANOVAs. This transformation is necessary because correlation coefficients do not follow a normal distribution, and are therefore strictly not amenable to analysis of variance statistics [19]. The Fisher z transformation converts correlation coefficients into normally distributed variables and thereby makes t-tests and ANOVAs possible. Given a correlation *r*, the Fisher z is given by

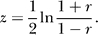



We took care to use the standard error formula specific to the Fisher z:

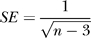



This formula has fewer degrees of freedom and is therefore more conservative than the conventional standard error.

### V1-like physical similarity measure.

To obtain a V1-like physical similarity measure, we applied a set of Gabor filters to the images and then computed pixelwise similarities between the images. This analysis was motivated by the fact that V1 cells exhibit tuning profiles that are well-described by Gabor filters [25,43]. Each image was convolved with Gabors of four different orientations (0°, 45°, 90°, and 135°; these orientations cover the whole unit circle because of the symmetry of the Gabors) and five different spatial frequencies (2, 4, 6, 8, and 10 cycles per degree). These parameters are representative of the tuning properties found in early visual cortex [25]. We then correlated the resulting physical similarity matrices with the neural similarities from our regions of interest, as described above; this was done both for the average across the V1-like similarities, as well as the individual matrices. The average V1-like physical similarity matrix correlated well (*r* = 0.67) with our other physical similarity measure, i.e. distance of the stimuli in parameter space.

## Abbreviations

IT: inferotemporal
FFA: fusiform face area
fMRI: functional magnetic resonance imaging
LO: posterior LOC
LOC: lateral occipital complex
OFA: occipital face area
pFs: anterior LOC
PPA: parahippocampal place area
ROI: region of interest

## References

[pbio-0060187-b001] Tanaka K, Saito H, Fukada Y, Moriya M (1991). Coding visual images of objects in the inferotemporal cortex of the macaque monkey. J Neurophysiol.

[pbio-0060187-b002] Malach R, Reppas JB, Benson RR, Kwong KK, Jiang H (1995). Object-related activity revealed by functional magnetic resonance imaging in human occipital cortex. Proc Natl Acad Sci U S A.

[pbio-0060187-b003] Kanwisher N, Woods R, Iacoboni M, Mazziotta J (1997). A locus in human extrastriate cortex for visual shape analysis. J Cog Neurosci.

[pbio-0060187-b004] James TW, Culham J, Humphrey GK, Milner AD, Goodale MA (2003). Ventral occipital lesions impair object recognition but not object-directed grasping: an fMRI study. Brain.

[pbio-0060187-b005] Grill-Spector K, Kushnir T, Hendler T, Malach R (2000). The dynamics of object-selective activation correlate with recognition performance in humans. Nat Neurosci.

[pbio-0060187-b006] Kourtzi Z, Kanwisher N (2001). Representation of perceived object shape by the human lateral occipital complex. Science.

[pbio-0060187-b007] Rotshtein P, Henson RN, Treves A, Driver J, Dolan RJ (2005). Morphing Marilyn into Maggie dissociates physical and identity face representations in the brain. Nat Neurosci.

[pbio-0060187-b008] Williams MA, Dang S, Kanwisher NG (2007). Only some spatial patterns of fMRI response are read out in task performance. Nat Neurosci.

[pbio-0060187-b009] Edelman S, Grill-Spector K, Kushnir T, Malach R (1998). Toward direct visualization of the internal shape representation space by fMRI. Psychobiology.

[pbio-0060187-b010] Kayaert G, Biederman I, Vogels R (2003). Shape tuning in macaque inferior temporal cortex. J Neurosci.

[pbio-0060187-b011] Kayaert G, Biederman I, Vogels R (2005). Representation of regular and irregular shapes in macaque inferotemporal cortex. Cereb Cortex.

[pbio-0060187-b012] Haxby JV, Gobbini MI, Furey ML, Ishai A, Schouten JL (2001). Distributed and overlapping representations of faces and objects in ventral temporal cortex. Science.

[pbio-0060187-b013] Spiridon M, Kanwisher N (2002). How distributed is visual category information in human occipito-temporal cortex? An fMRI study. Neuron.

[pbio-0060187-b014] Eger E, Ashburner J, Haynes JD, Dolan RJ, Rees G (2008). fMRI activity patterns in human LOC carry information about object exemplars within category. J Cogn Neurosci.

[pbio-0060187-b015] Logothetis NK, Pauls J, Augath M, Trinath T, Oeltermann A (2001). Neurophysiological investigation of the basis of the fMRI signal. Nature.

[pbio-0060187-b016] Kayaert G, Biederman I, Op de Beeck HP, Vogels R (2005). Tuning for shape dimensions in macaque inferior temporal cortex. Eur J Neurosci.

[pbio-0060187-b017] Op de Beeck HP, Baker CI, DiCarlo JJ, Kanwisher NG (2006). Discrimination training alters object representations in human extrastriate cortex. J Neurosci.

[pbio-0060187-b018] Grill-Spector K, Kourtzi Z, Kanwisher N (2001). The lateral occipital complex and its role in object recognition. Vision Res.

[pbio-0060187-b019] Fisher R (1915). Frequency distribution of the values of the correlation coefficient in samples of an indefinitely large population. Biometrika.

[pbio-0060187-b020] Kanwisher N, McDermott J, Chun MM (1997). The fusiform face area: a module in human extrastriate cortex specialized for face perception. J Neurosci.

[pbio-0060187-b021] Gauthier I, Tarr MJ, Moylan J, Skudlarski P, Gore JC (2000). The fusiform “face area” is part of a network that processes faces at the individual level. J Cogn Neurosci.

[pbio-0060187-b022] Kanwisher N, Yovel G (2006). The fusiform face area: a cortical region specialized for the perception of faces. Philos Trans R Soc Lond B Biol Sci.

[pbio-0060187-b023] Epstein R, Kanwisher N (1998). A cortical representation of the local visual environment. Nature.

[pbio-0060187-b024] Pasupathy A, Connor CE (1999). Responses to contour features in macaque area V4. J Neurophysiol.

[pbio-0060187-b025] Kay KN, Naselaris T, Prenger RJ, Gallant JL (2008). Identifying natural images from human brain activity. Nature.

[pbio-0060187-b026] Reddy L, Kanwisher N (2007). Category selectivity in the ventral visual pathway confers robustness to clutter and diverted attention. Curr Biol.

[pbio-0060187-b027] Op de Beeck H, Wagemans J, Vogels R (2001). Inferotemporal neurons represent low-dimensional configurations of parameterized shapes. Nat Neurosci.

[pbio-0060187-b028] Pasupathy A, Connor CE (2001). Shape representation in area V4: position-specific tuning for boundary conformation. J Neurophysiol.

[pbio-0060187-b029] Brincat SL, Connor CE (2004). Underlying principles of visual shape selectivity in posterior inferotemporal cortex. Nat Neurosci.

[pbio-0060187-b030] Wilkinson F, James TW, Wilson HR, Gati JS, Menon RS (2000). An fMRI study of the selective activation of human extrastriate form vision areas by radial and concentric gratings. Curr Biol.

[pbio-0060187-b031] Hayworth KJ, Biederman I (2006). Neural evidence for intermediate representations in object recognition. Vision Res.

[pbio-0060187-b032] Op de Beeck H, Vogels R (2000). Spatial sensitivity of macaque inferior temporal neurons. J Comp Neurol.

[pbio-0060187-b033] DiCarlo JJ, Maunsell JH (2003). Anterior inferotemporal neurons of monkeys engaged in object recognition can be highly sensitive to object retinal position. J Neurophysiol.

[pbio-0060187-b034] Logothetis NK, Pauls J (1995). Psychophysical and physiological evidence for viewer-centered object representations in the primate. Cereb Cortex.

[pbio-0060187-b035] Grill-Spector K, Kushnir T, Edelman S, Avidan G, Itzchak Y (1999). Differential processing of objects under various viewing conditions in the human lateral occipital complex. Neuron.

[pbio-0060187-b036] Lerner Y, Hendler T, Ben-Bashat D, Harel M, Malach R (2001). A hierarchical axis of object processing stages in the human visual cortex. Cereb Cortex.

[pbio-0060187-b037] Lerner Y, Hendler T, Malach R (2002). Object-completion effects in the human lateral occipital complex. Cereb Cortex.

[pbio-0060187-b038] Kourtzi Z, Erb M, Grodd W, Bulthoff HH (2003). Representation of the perceived 3-D object shape in the human lateral occipital complex. Cereb Cortex.

[pbio-0060187-b039] Koffka K (1935). Principles of Gestalt Psychology.

[pbio-0060187-b040] Brainard DH (1997). The Psychophysics Toolbox. Spat Vis.

[pbio-0060187-b041] Cox RW, Jesmanowicz A (1999). Real-time 3D image registration for functional MRI. Magn Reson Med.

[pbio-0060187-b042] Grill-Spector K, Kushnir T, Hendler T, Edelman S, Itzchak Y (1998). A sequence of object-processing stages revealed by fMRI in the human occipital lobe. Hum Brain Mapp.

[pbio-0060187-b043] Hubel DH, Wiesel TN (1959). Receptive fields of single neurones in the cat's striate cortex. J Physiol.

